# Resistance development characteristics of reared German cockroach (Blattodea: Blattellidae) to chlorpyrifos

**DOI:** 10.1038/s41598-021-83130-2

**Published:** 2021-02-10

**Authors:** Weiyuan Hou, Juanjuan Xin, Hui Lu

**Affiliations:** Laboratory of Molecular Vector Biology, Haidian Center for Disease Prevention and Control, No. 5 Xibeiwang 2nd Street, Haidian District, Beijing, 100094 People’s Republic of China

**Keywords:** Chemical biology, Zoology

## Abstract

Understanding the process of resistance development of German cockroach, *Blattella germanica* (L.), in detail is necessary to potentially delay the development of insecticides resistance by rotation or discontinuation of insecticides at the right time. In this study, we investigated the resistance development of the reared German cockroach to chlorpyrifos (CPF) for 23 generations from susceptible cockroaches. CPF 50% lethal dose (LD_50_) and resistance ratio of each generation cockroaches were determined. The CPF LD_50_ to each generation cockroaches was used as the insecticide selection pressure of this generation by topical application. The resistance development curve was depicted according to the CPF LD_50_ to all 23 generations of cockroaches. As a result, a highly resistant German cockroach cohort to CPF, which the resistance ratio was 21.63, was obtained after 23 generations’ selection. During the selection, the cockroaches developed low resistance from F1 to F5, moderate resistance from F6 to F12, and high resistance from F13 to F23. There was a rapid resistance increase every 5–7 generations. The resistance growing showed relatively slow from F1 to F11. The fastest growing phase of the resistance was from F12 to F20, in which accounted for more than 80% of the total resistance increase in 23 generations. The development of resistance to CPF tended to slow down from F21 to F23. These findings may provide a basis for the rational use of insecticides, delaying the development of resistance by rotation or discontinuation.

## Introduction

The German cockroach, *Blattella germanica* (L.), is a widespread pest lives exclusively in human environments, including households, residential areas, hospitals, restaurants, markets, vehicles and so on^1–3^. It is a worldwide distributed specie and commonly found in tropical and subtropical regions^4^. The German cockroach is not only an important disseminator of, but is also a reservoir for, a wide range of pathogens (bacteria, fungi, and protozoa)^5–10^. Besides being a vector of a wide variety of pathogens, the German cockroach can also impact human health through production of asthma and rhinitis-triggering allergens^11–13^. Exposure to cockroach allergens is one of the strongest risk factor for the development of asthma in urban populations, especially among inner-city children^14–16^.

Chlorpyrifos (CPF, O,O-diethyl O-3,5,6-trichloro-2-pyridylphosphorothioate) is an acetylcholinesterase (AChE) inhibitor. As a broad-spectrum organophosphorus insecticide, it is one of the most widely used in the world in control of a wide variety of insects, including fleas, wasps, bees, cockroaches, and agricultural pests^17–19^. Insecticides are typically convenient, fast acting and inexpensive; therefore, they are also widely used in large amount to control infestations of the German cockroach^20^. The German cockroach management relies extensively on insecticides application including of the CPF^21^. However, extensive and indiscriminate application of insecticides for the management of the German cockroach has resulted in the development of resistance^22–24^. The German cockroach has been reported to have developed resistance to 42 unique insecticide active ingredients in different documented cases worldwide and is ranked as the world’s No. 2 insecticide resistant urban pest^25^. Currently, widespread insecticide resistance has become a major obstacle to the effective management of the German cockroach. The mechanisms of insecticide resistance in the German cockroach including behavioral resistance, target site insensitivity, metabolic detoxification^25^. Enhanced metabolism is the major resistance mechanism in many strains of German cockroach. Enzymes involved in the detoxification of insecticides are either at a higher level or have enhanced activity in resistant German cockroaches. Additional resistance mechanisms such as *kdr*-type and *Rdl* mutation contributing toward pyrethroid and fipronil resistance, respectively^26,27^.

Understanding the process of resistance development in detail is necessary to potentially delay the development of insecticides resistance by rotation or discontinuation of insecticides at the right time. Unfortunately, the detailed process of resistance development to most insecticides including CPF is still scarce in the German cockroach. In this study, we investigated the resistance development of the German cockroach to CPF for 23 generations from the 5th instar nymphs of susceptible cockroaches, using CPF 50% lethal dose (LD_50_) as the insecticide selection pressure by topical application method. The resistance of each generation to CPF was determined and the resistance development curve was obtained. This may provide a basis for delay the development of CPF resistance by rotation or discontinuation.

## Results

We obtained a highly resistant German cockroach cohort to CPF, which the resistance ratio was 21.63, after 23 generations of selection from susceptible strain (SS) cockroaches. The susceptibility of control strain (CS) cockroaches to CPF treated only by acetone was not altered.

**Figure 1:**
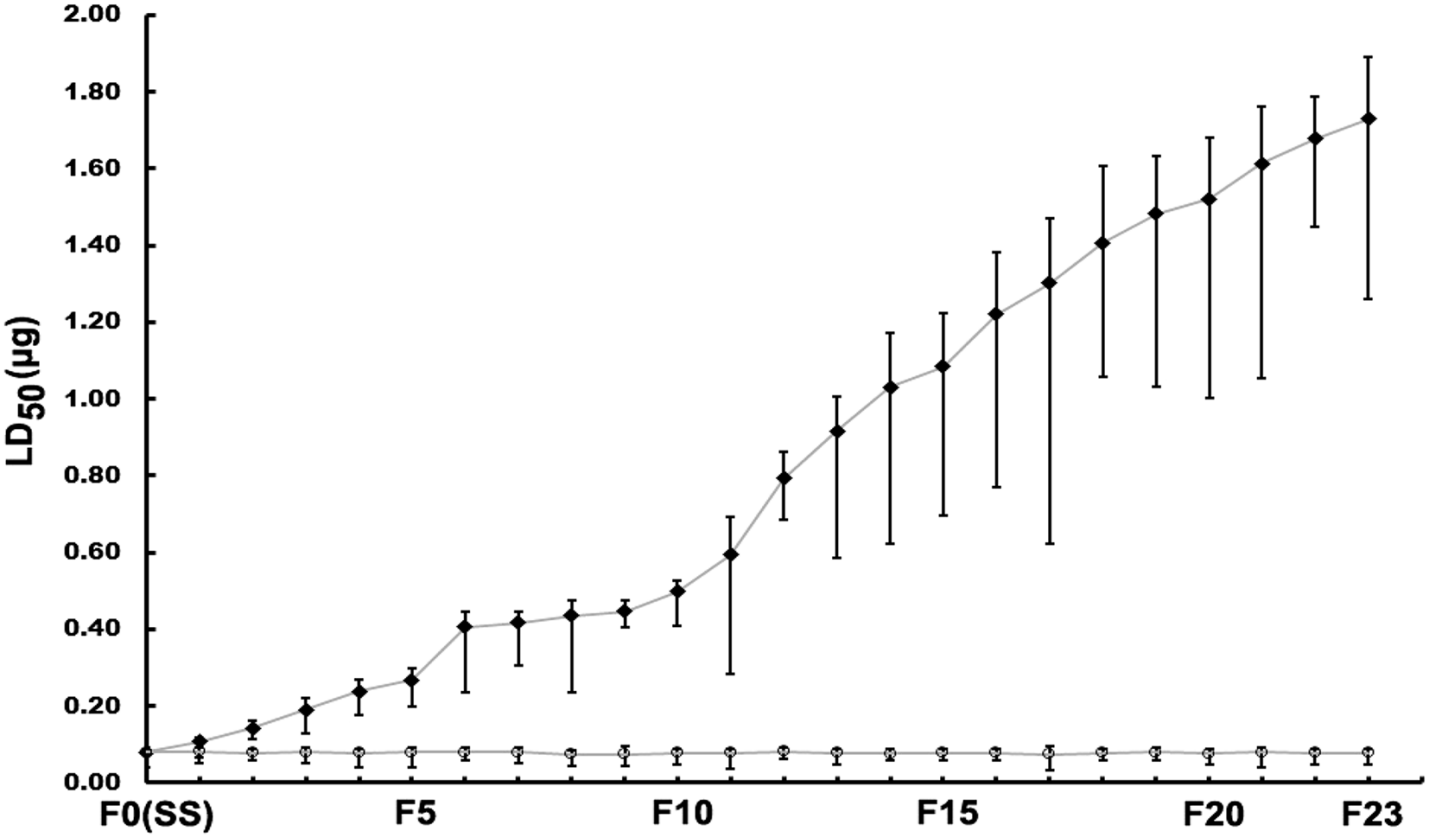
The LD_50_ development curve of German cockroach selected by CPF. O: control strain, ◆: CPF resistant strain. LD_50_: 50% lethal dose. Date were expressed as LD_50_ and the 95% confidence limit. Data were pooled from three independent experiments and subjected to regression/probit analysis using SPSS 13.0 to estimate the LD_50_ and 95% confidence limit.

The German cockroaches, after treated with CPF, developed low resistance from F1 to F5, moderate resistance from F6 to F12, high resistance from F13 to F23 (Table 1). A rapid resistance development every 5–7 generations was observed (from F5 to F6, from F11 to F12 and F15 to F16). The resistance of the German cockroaches to CPF developed relatively slow from F1 to F11, and the resistance level only increased to 7.38 fold. The fastest growing phase of the resistance to CPF was from F12 to F20, and the resistance ratio enhanced to 19.00, which accounted for more than 80% of the total resistance increase in 23 generations. The development of resistance to CPF tended to slow down from F21 to F23 (Fig. 1).

**Table 1:** Resistance development of German cockroaches from F0 to F23 generation selected by CPF.

Generation	Strain	Slope ± SE	Resistance ratio
F0	SS	7.90 ± 0.90	1.00
F1	CS	7.86 ± 0.88	1.00
	RS	8.91 ± 0.11	1.38
F2	CS	9.70 ± 1.42	1.00
	RS	7.00 ± 0.39	1.75
F3	CS	8.42 ± 1.32	1.00
	RS	7.17 ± 0.97	2.38
F4	CS	8.73 ± 0.57	1.00
	RS	7.13 ± 0.76	3.00
F5	CS	8.08 ± 0.63	1.00
	RS	8.20 ± 0.80	3.38
F6	CS	9.11 ± 1.83	1.00
	RS	11.52 ± 1.22	5.13
F7	CS	8.06 ± 1.20	1.00
	RS	11.20 ± 0.51	5.25
F8	CS	8.61 ± 1.56	1.00
	RS	11.47 ± 1.91	5.50
F9	CS	8.62 ± 1.49	0.88
	RS	15.35 ± 1.10	5.63
F10	CS	7.77 ± 0.35	1.00
	RS	15.83 ± 0.67	6.25
F11	CS	7.99 ± 1.51	1.00
	RS	6.78 ± 0.89	7.38
F12	CS	8.21 ± 0.79	1.00
	RS	11.92 ± 2.14	9.88
F13	CS	8.52 ± 0.96	1.00
	RS	11.95 ± 0.37	11.50
F14	CS	9.90 ± 1.98	1.00
	RS	11.61 ± 4.22	12.88
F15	CS	8.38 ± 1.17	1.00
	RS	10.25 ± 1.58	13.50
F16	CS	8.22 ± 1.31	1.00
	RS	9.44 ± 1.05	15.25
F17	CS	8.12 ± 1.35	0.88
	RS	8.10 ± 0.42	16.25
F18	CS	9.11 ± 1.28	1.00
	RS	9.62 ± 0.83	17.63
F19	CS	8.61 ± 1.86	1.00
	RS	11.54 ± 1.14	18.50
F20	CS	8.24 ± 0.27	1.00
	RS	10.49 ± 0.33	19.00
F21	CS	7.76 ± 0.83	1.00
	RS	12.40 ± 2.76	20.13
F22	CS	8.46 ± 0.22	1.00
	RS	14.13 ± 2.07	21.00
F23	CS	8.65 ± 1.64	1.00
	RS	11.86 ± 0.73	21.63

Data were pooled from three independent experiments and subjected to regression/probit analysis using SPSS 13.0 to estimate the slope, 50% lethal dose (LD_50_), then the mean and SE were calculated. Resistance ratio = LD_50_ (CS) or LD_50_ (RS)/ LD_50_ (SS).

*SS* susceptible strain, *CS* control strain, *RS* CPF resistant strain, *SE* standard error.

## Discussion

In this study, we followed the resistance development of the German cockroach to CPF for 23 generations. We obtained 21.63-fold resistance the German cockroaches to CPF, while the susceptibility of cockroaches to CPF treated only by acetone were not altered. This suggested that no other unexpected changes occur during 23 generations’ selection except treatment with CPF. The resistance development of the German cockroaches to CPF experienced three stages, which were the early slow rate development stage, the middle rapid development stage and the later slow down stage. The slow rate of resistance development in the early stage may reflect the initiation process of resistance related alleles such as various detoxification enzymes genes cytochrome P450 monooxygenases, carboxylesterases, glutathione transferases and so on^2,28,29^. It is possible that during selection, an accumulation of resistance factors such as detoxification enzymes systems leads to rapid detoxification of the insecticide or alteration of the sensibility of the insecticide target site AChE and preventing the insecticide-target site interaction occurred^30,31^. This may be the principal reason that the rapid resistance development in the middle stage. With the progress of screening, the changes of detoxification enzymes systems and insecticide target site have reached a plateau, therefore, the development of resistance is slowed down. Consequently, high resistance to CPF may have been developed as an evolutionary adaptation.

Insecticides are an essential part of an integrated pest management program. However, the extensive and intensive use associated with heavy reliance on insecticides have led to the development of resistance to the major groups of insecticide in many varieties of pests^25,26,32,33^. Rotation or discontinuation of insecticides is an important means to delay the development of resistance. According to the exact resistance development of the German cockroach to CPF together with its life cycle, the time of the insecticide rotation or discontinuation can be approximately inferred, so that the resistance will not develop to irreparable level. Our results showed that the German cockroach appeared moderate resistance in the F6 generation and high resistance in the F13 generation after CPF selection. This indicated that the CPF must be rotated or discontinued by the F13 generation. Under laboratory conditions, the generation cycle of the German cockroach is about 90 days, which means that it can probably reproduce four generations in a year^34^. The German cockroach may achieve high resistance if CPF is continuously used for about 3–4 years. These evidences may be useful for the use of insecticides in the field.

In summary, the resistance development of the reared German cockroach to CPF for 23 generations showed slow phase (F1–F11), fastest growing phase (F12–F20), slow down phase (F21–F23). There was a rapid resistance increase every 5–7 generations. This may have reference value for the rational use of insecticides in the field.

## Materials and methods

### Ethics statement

The German cockroaches used in this study were from our laboratory stock culture. The German cockroach is not an endangered or protected species. These cockroaches were only used for scientific research for public health purposes. All animal procedures were performed in accordance with current China legislation and approved by the Beijing Center for Disease Control and Prevention Animal and Medical Ethics Committee.

### Insecticide

CPF (purity > 98%) was obtained from China Vegetable Agriculture Development Co. Ltd (China, Beijing).

### Rearing of cockroaches

A standard German cockroach susceptible strain (SS) without exposure to any insecticide was obtain from the Institute of Microbiology and Epidemiology, Academy of Military Medical Sciences, Beijing, P.R. China. All SS, control strain (CS) and CPF-resistant (CPF-R) cockroaches were reared in glass cylinders maintained at 26 °C ± 1 °C, with a 12 h light: 12 h dark photoperiod (L12:D12), and a relative of humidity of 60 ± 10%, with mouse food and water ad libitum.

### Selection of CPF-R cockroaches

CPF-R cockroaches were selected from the 5th instar nymphs of SS cockroaches by topical application of CPF over 23 generations. In detail, 5th instar nymphs were selected from each generation. After anesthetizing with CO_2_, the LD_50_ of CPF for each generation was determined by topical bioassay. Each cockroach of every generation was given 1 μl of the LD_50_ dose of CPF dissolved in acetone between the first and second abdominal segments, using a microapplicator. CS cockroaches were treated with 1 μl of acetone alone. The treated cohorts of cockroaches were reared in separate glass cylinders with food and water ad libitum.

### Topical bioassay

Healthy male adult cockroaches selected from each generation were subjected to topical assays to generate an LD_50_ value as described in previous literatures^35–37^. A series of seven CPF doses that resulted mortality rates > 0% and < 100% were prepared. After anesthetizing with CO_2_, 1 μl of a given preparation was dropped on to the first and second abdominal segments of the cockroaches. Each concentration was applied to 20 adults with 3 replications. The treated cohorts of cockroaches were placed in separate glass cylinders and provided with food and water ad libitum. The survival of the cockroaches was recorded 24 h later.

### Data analysis

Bioassay data were pooled and subjected to probit analysis using SPSS 13.0 (Chicago, IL) to estimate the LD_50_ and LD_95_ values.
